# Beneficial biological effects and the underlying mechanisms of molecular hydrogen - comprehensive review of 321 original articles -

**DOI:** 10.1186/s13618-015-0035-1

**Published:** 2015-10-19

**Authors:** Masatoshi Ichihara, Sayaka Sobue, Mikako Ito, Masafumi Ito, Masaaki Hirayama, Kinji Ohno

**Affiliations:** Department of Biomedical Sciences, College of Life and Health Sciences, Chubu University, 1200 Matsumoto-cho, Kasugai, 487-8501 Japan; Division of Neurogenetics, Center for Neurological Diseases and Cancer, Nagoya University Graduate School of Medicine, 65 Tsurumai, Showa-ku Nagoya, 466-8550 Japan; Research Team for Mechanism of Aging, Tokyo Metropolitan Institute of Gerontology, 35-2 Sakae-cho, Itabashi, Tokyo, 173-0015 Japan; Department of Pathophysiological Laboratory Sciences, Nagoya University Graduate School of Medicine, 1-1-20 Daiko-Minami, Higashi-ku, Nagoya, 461-8673 Japan

**Keywords:** Molecular hydrogen, Ischemia-reperfusion injury, Inflammatory diseases

## Abstract

Therapeutic effects of molecular hydrogen for a wide range of disease models and human diseases have been investigated since 2007. A total of 321 original articles have been published from 2007 to June 2015. Most studies have been conducted in Japan, China, and the USA. About three-quarters of the articles show the effects in mice and rats. The number of clinical trials is increasing every year. In most diseases, the effect of hydrogen has been reported with hydrogen water or hydrogen gas, which was followed by confirmation of the effect with hydrogen-rich saline. Hydrogen water is mostly given *ad libitum*. Hydrogen gas of less than 4 % is given by inhalation. The effects have been reported in essentially all organs covering 31 disease categories that can be subdivided into 166 disease models, human diseases, treatment-associated pathologies, and pathophysiological conditions of plants with a predominance of oxidative stress-mediated diseases and inflammatory diseases. Specific extinctions of hydroxyl radical and peroxynitrite were initially presented, but the radical-scavenging effect of hydrogen cannot be held solely accountable for its drastic effects. We and others have shown that the effects can be mediated by modulating activities and expressions of various molecules such as Lyn, ERK, p38, JNK, ASK1, Akt, GTP-Rac1, iNOS, Nox1, NF-κB p65, IκBα, STAT3, NFATc1, c-Fos, and ghrelin. Master regulator(s) that drive these modifications, however, remain to be elucidated and are currently being extensively investigated.

## Introduction

It has been 8 years since Ohsawa and colleagues reported the astonishing therapeutic effects of molecular hydrogen on a rat model of cerebral infarction in *Nature Medicine* in 2007 [1]. Inhalation of 1–4 % hydrogen gas markedly reduced the sizes of cerebral infarction in rats. They also demonstrated that hydrogen specifically scavenges hydroxyl radical and peroxynitrite but not hydrogen peroxide or superoxide. Their paper ignited interest in the effect of molecular hydrogen in various diseases and has been cited 533 times as of July 2015. Similarly, the number of original articles demonstrating the effect of molecular hydrogen adds up to more than 300. This review summarizes research articles published in these past 8 years and addresses possible molecular mechanisms underlying the effects of hydrogen.

### Molecular hydrogen research before 2007

Even before the publication by Ohsawa and colleagues in 2007 [1], biological effects of molecular hydrogen had been investigated in a small scale, as shown below. Dole and colleagues first reported the hydrogen effect in *Science* in 1975 [2]. They placed nude mice carrying squamous cell carcinoma in a chamber with 2.5 % oxygen and 97.5 % hydrogen under 8-atmospheric pressure and observed prominent reduction in the size of the tumors. A similar effect of hyperbaric hydrogen on leukemia was reported in 1978 [3]. Hydreliox, which contained 49 % hydrogen, 50 % helium, and 1 % oxygen, was reported to be effective to prevent decompression sickness and nitrogen narcosis for divers working below 500 meters under sea level [4]. An anti-inflammatory effect of hyperbaric hydrogen on a mouse model of schistosomiasis-associated chronic liver inflammation was also reported in 2001 [5]. Hyperbaric hydrogen may be effective for some diseases, but only a limited number of studies have been published. The difference between hyperbaric and normobaric hydrogen has not been directly compared to date.

Following a small number of studies with hyperbaric hydrogen, the effect of electrolytically alkaline water has been reported. Shirahata and colleagues hypothesized that the hydrogen atom, which they called active hydrogen, is generated in electrolysis and proposed that active hydrogen scavenges reactive oxygen species (ROS) [6]. Although it is unlikely that atomic hydrogen is able to exist for a substantial time in our bodies, molecular hydrogen does exist in electrolyzed water and the effects of electrolyzed water have been reported thereafter. Li and colleagues reported that electrolyzed water scavenged ROS and protected a hamster pancreatic beta cell line from alloxan-induced cell damage [7]. Similarly, reduced hemodialysis solution produced by an electrolysis device (Nihon Trim Co. Ltd.) ameliorated oxidative stress in hemodialysis patients [8]. In 2005, researchers in Tohoku University Graduate School of Medicine and Nihon Trim started cooperative clinical studies and established the Association of Electrolyzed Water-Hemodialysis Study Group in 2008. According to personal communications with this group, they now believe that the effects of electrolyzed water are likely due to dissolved hydrogen molecules.

In 2005, Yanagihara and colleagues at Miz Co. Ltd. reported that hydrogen-rich neutral water that was produced with their unique electrolysis device reduced oxidative stress in rats [9]. This was a pioneering work, because they explicitly proved that molecular hydrogen but not alkaline in the electrolyzed alkaline water exerts therapeutic effects.

### Molecular hydrogen research in and after year 2007

**Fig. 1:**
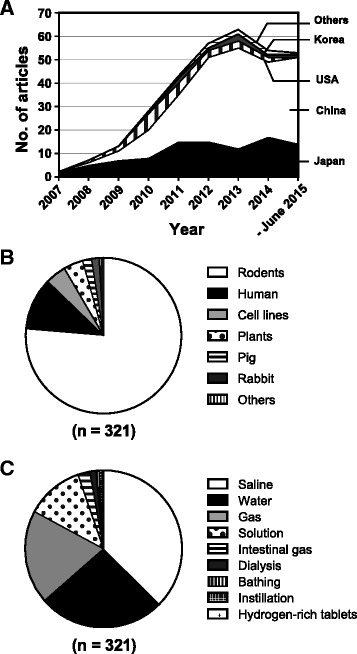
Profiles of 321 original articles up to June 2015 showing therapeutic effects of molecular hydrogen. **a** Temporal profile of countries where the studies are reported from 2007 to June 2015. **b** Biological species used in the studies. **c** Modalities of hydrogen administration to model animals, humans, and plants

As stated in the introduction, the *Nature Medicine* paper in 2007 [1] spurred interest in hydrogen research. Figure 1 shows 321 original articles up to June 2015 in the MEDLINE database, which demonstrate the effects of molecular hydrogen on disease models, human diseases, treatment-associated pathologies, and pathophysiological conditions of plants. Most studies were conducted in Japan, China, and the USA, with a predominance of China since 2010 (Fig. 1A). About three-quarters of the articles show the effects in mice and rats (Fig. 1B), but the number of human studies is increasing every year (1 article each in 2008–2009; 2 in 2010; 3 in 2011; 5 in 2012; 9 in 2013; 6 in 2014; and 6 in 2015). In addition, the effects of hydrogen have been reported in plants in 13 articles, which suggest a wide range of effects over various species not restricted to mammals. The effects of molecular hydrogen on plants may warrant application of hydrogen to increase agricultural production. Modalities of hydrogen administration are shown in Fig. 1C. Hydrogen-rich saline, which is almost exclusively used in China, dominates over the others. Hydrogenized saline is administered either by intraperitoneal injection or drip infusion. Hydrogen water is mostly given *ad libitum*. Hydrogen gas is usually given by inhaling 1–4 % hydrogen gas, which is below the explosion level (4 %). There is a single report, in which hydrogen gas was injected intraperitoneally [10].

**Fig. 2:**
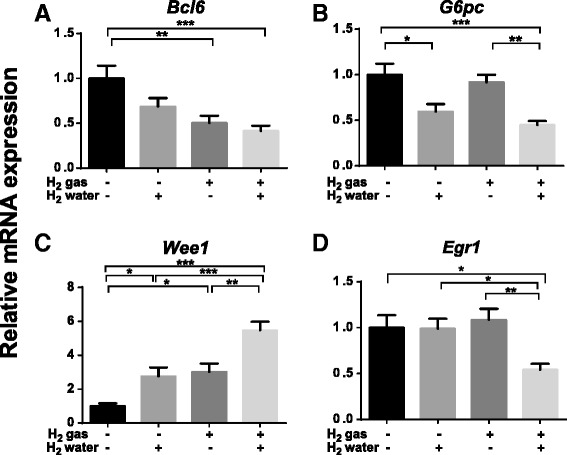
Four groups of genes that show different responses to hydrogen gas and/or water [12] . **a** *Bcl6* responds to hydrogen gas more than hydrogen water. **b** *G6pc* responds only to hydrogen water. **c** *Wee1* responds to both hydrogen water and gas. **d** *Egr1* responds only to simultaneous administration of hydrogen gas and water

Among the various routes of hydrogen administration shown in Fig. 1C, the best method still remains uncertain. This is partly because only a few reports have addressed the difference of effects among administration methods. We previously showed that drinking hydrogen water, but not continuous hydrogen gas exposure, prevented development of 6-hydorxydopamine-induced Parkinson’s disease in rats [11]. In addition, we recently showed that continuous exposure to hydrogen gas and *ad libitum per os* administration of hydrogen water modulated signaling pathways and gene expressions in different manners in mice [12]. We demonstrated that hydrogen-responsive genes are divided into four groups: genes that respond favorably to hydrogen gas, those that respond exclusively to hydrogen water, those that respond to both hydrogen gas and water, and those that respond only to the simultaneous administration of gas and water (Fig. 2). As hydrogen gas and water increase the hydrogen concentrations in the rodent body to a similar level [12], the difference in the organs exposed to a high concentration of hydrogen, the rise time of hydrogen concentration, and/or the area under the curve of hydrogen concentration may account for the difference in the modulated genes. On the other hand, a collation of hydrogen reports indicate that a similar degree of effects can be observed with different modalities of administration. For example, the marked effect of hydrogen on a mouse model of LPS-induced acute lung injury has been reported by four different groups with three different modalities: hydrogen gas [13, 14], hydrogen water [15], and hydrogen-rich saline [14, 16]. Similarly, the dramatic effect of hydrogen on animal models of acute myocardial infarction has been reported by eight different groups with two different modalities: hydrogen gas [17–20] and hydrogen-rich saline [21–24]. To clarify the difference of hydrogen’s effects with different modalities of administration, each research group should scrutinize the difference of the effects between hydrogen gas, hydrogen water, and hydrogen-rich saline. This would uncover the best modality for each disease model, if any, and also the optimal hydrogen dose.

Table 1 summarizes disease categories for which the effects of hydrogen have been reported. Ohsawa and colleagues reported the hydrogen effect in cerebral infarction [1] and many subsequent studies also showed its effect in ischemia-reperfusion injuries including organ transplantations. Following the initial report by Ohsawa and colleagues, the specific hydroxyl radical scavenging effect of hydrogen has been repeatedly proposed in oxidative stress-mediated diseases including inflammatory diseases and metabolic diseases.

**Table 1:** Disease categories for which hydrogen exhibited beneficial effects

Pathophysiology	No. of articles	%
Oxidative stress	224	69.8
(I/R injury	80	24.9)
(Others	144	44.9)
Inflammation	66	20.6
Metabolism	20	6.2
Others	11	3.4

*I/R* ischemia/reperfusion

Table 2 shows the details of organs and diseases for which the effects of hydrogen have been reported. Table 2 is an update of our previous review article in 2012 [25]. We have now classified the organs and diseases into 31 categories and showed the effects in 166 disease models, human diseases, treatment-associated pathologies, and pathophysiological conditions of plants. Hydrogen is effective in essentially all organs, as well as in plants.

**Table 2:** Disease models, human diseases, treatment-associated pathologies, and pathophysiological conditions of plants (321 original articles published in English) for which the effects of hydrogen have been reported from 2007 to June 2015

Diseases and conditions	References
Brain	
*Cerebrovascular diseases (CVD)*	
Cerebral I/R injury	[1, 10, 56, 83, 94, 99–109]
Hypertensive stroke	[110]
Brain injury secondary to intracerebral hemorrhage	[28]
Subarachnoid hemorrhage	[48, 61, 66, 73, 111–113]
*Brain injury other than CVD*	
Traumatic brain injury	[114–118]
Deep hypothermic circulatory arrest-induced brain damage	[57]
*Neurodegenerative diseases*	
Parkinson’s disease	[11, 95–97, 119]
Alzheimer’s disease	[43, 120]
*Others*	
Restraint-induced dementia	[121]
Senile dementia in senescence-accelerated mice	[122]
LPS-induced neuroinflammation	[81, 123]
Oxidative stress-induced neuronal cell damage	[124, 125]
Spinal Cord and peripheral nerve	
Spinal cord I/R injury	[126, 127]
Spinal cord injury	[77, 128]
Neuropathic pain	[39, 92, 129, 130]
Hyperalgesia	[79, 131, 132]
Eye	
Retinal I/R injury	[133, 134]
Diabetic retinopathy	[135, 136]
Hyperoxia-induced retinopathy	[137]
Light-induced retinopathy	[138, 139]
Glutamine-induced retinopathy	[50]
S-nitroso-N-acetylpenicillamine-induced retinopathy	[140]
Optic nerve crush	[141]
Selenite-induced cataract	[142]
Corneal alkali-burn	[55]
Anti-inflammatory effects on LPS-activated retinal microglia cells	[64]
Ear	
Hearing loss	[143–148]
Cisplatin-induced ototoxicity	[149, 150]
Ouabain-induced ototoxicity	[151]
Oral Cavity	
Periodontitis	[32]
Periodontal oxidative damage	[152]
Lung	
Lung I/R injury	[153, 154]
Oxygen-induced lung injury	[82, 155, 156]
Ventilation-induced lung injury	[53, 157]
LPS-induced acute lung injury	[13, 14, 16, 158]
Intestinal I/R-induced lung injury	[159]
Burn-induced lung injury	[160]
Paraquat-induced lung injury	[161, 162]
igarette smoking lung injury	[163]
Smoke inhalation lung injury	[74]
Pulmonary hypertension	[78, 164]
Heart	
Myocardial infarction and I/R injury	[17–24, 84]
Diabetic cardiomyopathy	[40]
Sleep apnea-induced left ventricular remodeling	[165, 166]
Ventricular hypertrophy	[167]
Stomach	
Stress-induced gastric ulceration	[38]
Aspirin-induced gastric ulceration	[168, 169]
Intestine	
Intestinal I/R injury	[170, 171]
Ulcerative colitis	[172, 173]
Colon inflammation	[174]
Sepsis-induced intestinal injury	[87]
Necrotizing enterocolitis	[175]
Liver	
Liver I/R injury	[71, 98, 176–178]
Chronic hepatitis B	[179]
Nonalcoholic steatohepatitis	[180]
Liver injury induced by massive hepatectomy	[67, 93, 181]
Liver injury induced by obstructive jaundice	[31]
Liver injury induced by endotoxin	[35]
Liver injury induced by acetaminophen	[47]
Liver injury induced by carbon tetrachloride	[42]
Liver injury induced by concanavalin A	[182]
Liver cirrhosis	[183]
Liver fibrosis	[184]
Pancreas	
Acute pancreatitis	[76, 185–187]
Peritoneum	
Acute peritonitis	[68]
Kidney	
Renal I/R injury	[188–190]
Acute renal injury	[37, 72, 191–194]
Hypertensive renal injury	[69]
Cisplatin-induced nephropathy	[195–197]
Gentamicin-induced nephrotoxicity	[198]
Inhibition of AGEs production	[199]
Renal calcium deposition	[200]
Bladder	
Interstitial cystitis	[201]
Reproductive organ	
Testicular I/R injury	[202, 203]
Erectile dysfunction	[204]
Nicotine-induced testicular oxidative stress	[205]
Cigarette smoke-induced testicular damage	[206]
Skin	
I/R injury	[46, 207]
UV-induced skin injury	[45, 208–211]
Acute erythematous skin disease	[212]
Atopic dermatitis	[213, 214]
Psoriasis	[215]
Pressure ulcer	[216]
Burn	[49, 70]
Arsenic toxicity	[217]
Bone and Joint	
Rheumatoid arthritis	[218, 219]
Osteoporosis	[36, 62]
Bone loss induced by microgravity	[34]
TNFα-induced osteoblast injury	[220]
NO-induced cartilage toxicity	[221]
Skeletal Muscle and soft tissue	
I/R injury in skeletal muscle	[222]
Inflammatory and mitochondrial myopathies	[223]
Muscle fatigue	[224]
Sports-related soft tissue injury	[225]
Blood vessel	
Atherosclerosis	[58, 59, 85, 226, 227]
AGEs-induced blood vessel damage	[228]
Neointimal hyperplasia	[29]
Hyperplasia in arterialized vein graft	[229]
Vascular dysfunction	[60]
Vascular endothelial function	[230]
Blood and Bone Marrow	
Aplastic anemia	[231]
Maintenance of multipotential stroma/mesenchymal stem cells	[232]
Neutrophil function	[233]
Inhibition of collagen-induced platelet aggregation	[234]
Improvement of blood fluidity	[235]
Metabolism	
Diabetes mellitus	[236–241]
Hyperlipidemia	[44, 242–244]
Metabolic syndrome	[245–247]
Metabolic process-related gene expression	[248]
Oxidized low density lipoprotein-induced cell toxicity	[54]
Serum alkalinization	[249]
Exercise-induced metabolic acidosis	[250]
Inflammation/Allergy	
Sepsis	[41, 86, 251–255]
LPS/IFNγ-induced NO production	[27]
LPS-induced inflammatory response	[90]
LPS-induced vascular permeability	[80, 256]
Zymosan-induced inflammation	[257]
Carrageenan-induced paw edema	[258]
Inflammatory response of cardiopulmonary bypass	[259]
Type I allergy	[26]
Asthma	[63]
Perinatal Disorders	
Neonatal cerebral hypoxia	[260–263]
LPS-induced fetal lung injury	[15]
Preeclampsia	[264, 265]
Cancer	
Growth of tongue carcinoma cells	[266]
Fe-NTA-induced nephrotoxicity and tumor progression	[65]
Radiation-induced thymic lymphoma	[267]
Tumor angiogenesis	[268]
Enhancement of 5-FU antitumor efficacy	[269]
Radiation	
Cardiac damage	[270]
Lung damage	[271]
Testicular damage	[272]
Skin damage	[273, 274]
Germ, hematopoietic and other cell damage	[275–280]
Radiation-induced adverse effects	[281]
Radiation-induced immune dysfunction	[282]
Intoxication	
Carbon monoxide	[283–286]
Sevoflurane	[287, 288]
Doxorubicin-induced heart failure	[289]
Melamine-induced urinary stone	[290]
Chlorpyrifos-induced neurotoxicity	[291]
Transplantation	
Heart	[52, 292–294]
Lung	[33, 88, 295–299]
Kidney	[30, 51]
Intestine	[89, 300, 301]
Pancreas	[302]
Osteochondral grafts	[303]
Acute GVHD	[304, 305]
Resuscitation	
Cardiac arrest	[306, 307]
Hemorrhagic shock	[75, 308, 309]
Dialysis	
Hemodialysis	[310–313]
Peritoneal dialysis	[314, 315]
Others	
Lifespan extension	[316]
Sperm motility	[317]
Decompression sickness	[318]
Genotoxicity and mutagenicity	[319]
Plant	
Root organogenesis	[91, 320]
Salt tolerance	[321, 322]
Postharvest ripening	[323]
Stomatal closure	[324]
Radish sprout tolerance to UVA	[325]
High light stress	[326]
Phytohormone signaling and stress responses	[327]
Tolerance to paraquat-induced oxidative stress	[328]
Cadmium toxicity	[329, 330]
Mercury toxicity	[331]

### Molecular mechanisms of the effects of hydrogen

Collation of the 321 original articles reveals that most communications address the anti-oxidative stress, anti-inflammatory, and anti-apoptotic effects. Specific scavenging activities of hydroxyl radical and peroxynitrite, however, cannot fully explain the anti-inflammatory and anti-apoptotic effects, which should involve a number of fine-tuned signaling pathways. We have shown that hydrogen suppresses signaling pathways in allergies [26] and inflammation [27] without directly scavenging reactive oxygen/nitrogen species. Signaling molecules that are modulated by hydrogen include Lyn [26, 28], Ras [29], MEK [29, 30], ERK [12, 24, 29–37], p38 [12, 16, 24, 27, 30, 32, 33, 35–41], JNK [13, 24, 27, 30, 32, 33, 35–38, 40, 42–47], ASK1 [27, 46], Akt [12, 29, 36, 37, 48, 49], GTP-Rac1 [36], iNOS [27, 34, 36, 50–52], Nox1 [36], NF-κB p65 or NF-κB [12, 14, 27, 35–38, 40, 41, 43, 49, 53–75], IκBα [27, 40, 41, 54, 60, 62, 69, 73, 76], STAT3 [65, 77, 78], NFATc1 [12, 36, 78], c-Fos [36], GSK-3β [48, 79], ROCK [80]. Activities and expressions of these molecules are modified by hydrogen. Master regulator(s) that drive these modifications remain to be elucidated.

The anti-oxidative stress effect of hydrogen was first reported to be conferred by direct elimination of hydroxyl radical and peroxynitrite. Subsequent studies indicate that hydrogen activates the Nrf2-Keap1 system. Hydrogen activates Nrf2 [36, 81–87] and its downstream heme oxygenase-1 (HO-1) [36, 51, 52, 65, 71, 81, 82, 84–93]. Kawamura and colleagues reported that hydrogen did not mitigate hyperoxic lung injury in *Nrf2*-knockout mice [82]. Similarly, Ohsawa and colleagues reported that hydrogen enhanced mitochondrial functions and induced nuclear translocation of Nrf2 at the Symposium of Medical Molecular Hydrogen in 2012 and 2013. They proposed that hydrogen induces an adaptive response against oxidative stress, which is also known as a hormesis effect. These studies indicate that the effect of hydrogen is mediated by Nrf2, but the mechanisms of how Nrf2 is activated by hydrogen remain to be solved.

Another interesting mechanism is that hydrogen modulates miRNA expressions [64, 94]. Hydrogen regulates expressions of miR-9, miR-21, and miR-199, and modifies expressions of IKK-β, NF-κB, and PDCD4 in LPS-activated retinal microglia cells [64]. Similarly, analysis of miRNA profiles of hippocampal neurons during I/R injury revealed that hydrogen inhibits I/R-induced expression of the miR-200 family by reducing ROS production, which has led to suppression of cell death [94]. However, modulation of miRNA expression cannot solely explain all the biological effects mediated by hydrogen. In addition, mechanisms underlying modulated miRNA expressions remain to be elucidated.

Matsumoto and colleagues reported that oral intake of hydrogen water increased gastric expression and secretion of ghrelin and that the neuroprotective effect of hydrogen water was abolished by the ghrelin receptor-antagonist and by the ghrelin secretion-antagonist [95]. As stated above, we have shown that hydrogen water, but not hydrogen gas, prevented development of Parkinson’s disease in a rat model [11]. Prominent effect of oral hydrogen intake rather than hydrogen gas inhalation may be partly accounted for by gastric induction of ghrelin.

**Fig. 3:**
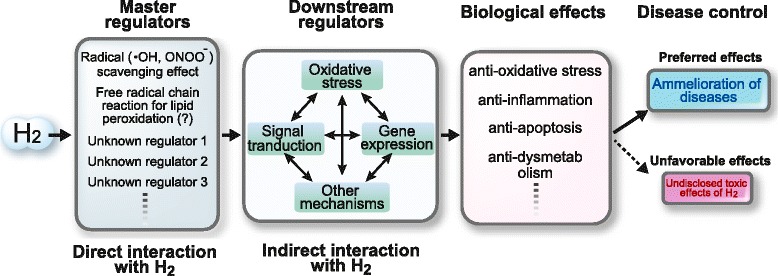
Schematic summary of molecular mechanisms of hydrogen

Recently, Ohta and colleagues showed at the 5th Symposium of Medical Molecular Hydrogen at Nagoya, Japan in 2015 that hydrogen influences a free radical chain reaction of unsaturated fatty acid on cell membrane and modifies its lipid peroxidation process. Furthermore, they demonstrated that air-oxidized phospholipid that was produced either in the presence or absence of hydrogen *in vitro*, gives rise to different intracellular signaling and gene expression profiles when added to the culture medium. They also showed that this aberrant oxidization of phospholipid was observed with a low concentration of hydrogen (at least 1.3 %), suggesting that the biological effects of hydrogen could be explained by the aberrant oxidation of phospholipid under hydrogen exposure. Among the many molecules that are altered by hydrogen, most are predicted to be passengers (downstream regulators) that are modulated secondarily to a change in a driver (master regulator). The best way to identify the master regulator is to prove the effect of hydrogen in an *in vitro* system. Although, to our knowledge, the study on lipid peroxidation has not yet been published, the free radical chain reaction for lipid peroxidation might be the second master regulator of hydrogen next to the radical scavenging effect. We are also analyzing other novel molecules as possible master regulators of hydrogen (in preparation). Taken together, hydrogen is likely to have multiple master regulators, which drive a diverse array of downstream regulators and achieve beneficial biological effects against oxidative stress, inflammation, apoptosis, and dysmetabolism to name a few (Fig. 3).

These studies all point to the notion that hydrogen modulates intracellular signal transduction systems and regulates the downstream gene expressions to mitigate disease processes. In general, biologically active substances that modulate signaling molecules have both beneficial and noxious effects on our bodies. Hydrogen may also have undisclosed toxic effects, although none have been reported to date to the best of our knowledge. Understanding the exact molecular mechanisms of the effects of hydrogen will elucidate its master regulator(s) and clarify the pros and cons of hydrogen therapy, which will also potentially lead to the development of another therapeutic modality to modulate the master regulator(s). We summarized in Table 3 original articles that addressed biological effects and *in vivo* kinetics of hydrogen, which were not directly relevant to disease models or human diseases. It is essential to elucidate detailed pharmacokinetics of hydrogen *in vivo* from the viewpoint of clinical application of hydrogen, although we have accumulated vast knowledge about the effects and not the kinetics of hydrogen in disease models and human diseases. Through these analyses, promising outcomes are expected for more effective administration regimen of hydrogen therapy.

**Table 3:** Original articles showing physiological effects and *in vivo* kinetics of hydrogen

Biological effects and *in vivo* kinetics of hydrogen	References
Superoxide formation in brain slices in mice	[332]
Gene expression profiles and signal transduction pathways evaluated by DNA microarray and RNA-seq in rodents	[33]^a^, [12], [118]^a^, [248]^a^
Comparison of intermittent and continuous administration of hydrogen gas in rats	[11]^a^
Safety of hydrogen inhalation in patients with cerebral ischemia	[333]
A convenient method to estimate the concentration of hydrogen in water	[334]
Hydrogen consumption in human body after hydrogen administration	[335, 336]
Ghrelin induction and secretion by hydrogen-dissolved water in mice	[95]^a^
Additive effects of hydrogen and NO	[20, 158]^a^
*In vivo* kinetics of hydrogen after hydrogen administration in rodents	[12, 337]
Lack of reactivity of hydrogen with peroxynitrite	[338]
Antioxidant activity of nano-bubble hydrogen-dissolved water	[339]
Additive effects of hydrogen gas and hydrogen-rich water	[12]

^a^These articles are also listed in Table 2

### Clinical studies of molecular hydrogen

As stated in the introduction, the number of clinical trials has been increasing since 2011. About half of human studies have been conducted in Japan. Dependable studies recruiting more than ten patients or employing double-blind studies are summarized in Table 4.

**Table 4:** Clinical trials published as of June, 2015

Authors/Year	Disease	Sample size	Open-label (OL), double-blind (DB), or single-blind (SB)	Hydrogen administration	Summary of the outcome
Kajiyama et al. [236]/2008	Diabetes mellitus type II	30	DB	Water	Improvement of fractions of low-density lipoprotein (LDL)-cholesterol and a glucose tolerance test.
Nakao et al. [245]/2010	Metabolic syndrome	20	OL	Water	Improvement of urinary markers for oxidative stress such as SOD and TBARS, and increase of high-density lipoprotein (HDL)-cholesterol.
Nakayama et al. [311]/2010	Chronic renal failure	29	OL	Dialysis	Amelioration of hypertension and improvement of markers for oxidative stress and inflammation.
Ito et al. [223]/2011	Inflammatory and mitochondrial myopathies	31	OL/DB	Water	OL: Improvement of the serum lactate/pyruvate ratio in mitochondrial myopathies and the serum matrix metalloproteinse-3 level in polymyositis/dermatomyositis.
					DB: Improvement of the serum lactate.
Kang et al. [281]/2011	Radiation-induced adverse effects for liver tumors	49	OL	Water	Improvement of quality of life (QOL) scores during radiotherapy.
					Reduction of blood reactive oxygen metabolites and maintenance of blood oxidation potential.
Ishibashi et al. [218]/2012	Rheumatoid arthritis	20	OL	Water	Improvement of disease activity score for rheumatoid arthritis (DAS28).
					Decrease of urinary 8-OHdG.
Aoki et al. [224]/2012	Muscle fatigue	10	DB	Water	Improvement of muscle fatigue in young athletes
Li et al. [216]/2013	Pressure skin ulcer	22	OL	Water	Wound size reduction and early recovery from skin pressure ulcer.
Matsumoto et al. [201]/2013	Interstitial cystitis	30	DB	Water	No significant effect on symptoms.
					Reduction of the bladder pain score in 11 % of patients.
Nagatani et al. [106]/2013	Cerebral ischemia	38	OL	Intravenous infusion	Confirmation of safety of intravenous H_2_ infusion.
					Decrease of MDA-LDL, a serum marker for oxidative stress, in a subset of patients.
Shin et al. [45]/2013	UV-induced skin injury	28	OL	Gas	Prevention and modulation of UV-induced skin inflammation, intrinsic skin aging, and photo aging process through reduction of MMP-1, IL-6, and IL-1b mRNA expression.
Song et al. [243]/2013	Hyperlipidemia	20	OL	Water	Decrease of total serum cholesterol, LDL-cholesterol, apolipoprotein (apo) B100, and apoE
Xia et al. [179]/2013	Chronic hepatitis B	60	DB	Water	Attenuation of oxidative stress
Yoritaka et al. [96]/2013	Parkinson disease	17	DB	Water	Improvement of Total Unified Parkinson’s Disease Rating Scale (UPDRS) and exacerbation after termination of H_2_ water.
Ishibashi et al. [219]/2014	Rheumatoid arthritis	24	DB	Intravenous saline infusion	Improvement of DAS28.
					Decrease of serum IL-6, MMP3, CRP, and urinary 8-OHdG.
Ostojic et al. [225]/2014	Sports-related soft tissue injury	36	SB	H2-rich tablets and topical H_2_ packs	Decrease of plasma viscosity.
					Faster recovery from soft tissue injury.
Ostojic et al. [250]/2014	Exercise-induced metabolic acidosis	52	DB	Water	Increased blood alkalinity in physically active men.
Sakai et al. [230]/2014	Vascular endothelial function.	34	DB	Water	Increased flow-mediated dilation of branchial artery, suggesting that H_2_ can serve as a modulator of vasomotor function of vasculature.
Song et al. [244]/2015	Hyperlipidemia	68	DB	Water	Down-regulation of plasma levels of total cholesterol, and LDL-cholesterol, followed by increased plasma pre-β -HDL, apoM, and decreased plasma oxidized-LDL, apoB100.

Features shared in these clinical studies are that hydrogen exhibits statistically significant effects in patients but the effects are usually not as conspicuous as those observed in rodent models. These can be accounted for by i) the difference in species, ii) technical difficulty in preparing a high concentration of hydrogen water every day for the patients, and iii) the difference between acute and chronic diseases. Further large-scale and long-term clinical studies are expected to prove the effects of hydrogen in humans.

Table 5 shows clinical studies currently registered in Japan. Researchers in Juntendo University have started a large-scale clinical trial of Parkinson’s disease after they have shown the effects of molecular hydrogen in a small number of patients in a short duration [96]. Being prompted by the prominent effects of hydrogen for mouse models with ischemia reperfusion injuries, clinical trials for acute post cardiac arrest syndrome and myocardial infarction have started at Keio University. Similarly, a clinical trial for cerebral infarction has started at the National Defense Medical College.

**Table 5:** Clinical trials registered in Japan as of June, 2015

Date	Disease	Affiliation	Status
7/16/2008	Interstitial cystitis	Koshinkai Hosp.	Finished[201]
8/21/2008	Impaired glucose tolerance and impaired fasting glycaemia	Digestive tract internal medicine, Kyoto Prefectural University of Medicine	Finished [236].
7/17/2009	Mild cognitive impairment	Neuropsychiatry, Tsukuba Univ.	Finished
1/11/2011	Chronic hemodialysis	Nephrology, Fukushima Medical University	Trial in progress
6/2/2011	Acute cerebral infarction	Neurosurgery, Self Defense Medical College	Calling for participants[106]
9/30/2011	Normal adults	Faculty of Health Sciences, Kyorin Univ.	Finished
12/4/2011	Acute myocardial infarction	Cardiology, Keio Univ.	Calling for participants
3/14/2012	Parkinson’s disease	Neurology, Juntendo Univ.	Finished [96]
10/16/2012	Multiple system atrophy, Progressive supranuclear palsy	Neurology, Juntendo Univ.	Trial in progress
2/13/2013	Parkinson’s disease	Neurology, Juntendo Univ.	Calling for participants
5/1/2013	Chronic obstructive pulmonary disease	Respiratory Medicine, Juntendo Univ.	Trial in progress
5/20/2013	Hepatitis and liver cirrhosis	Gastroenterology and Hepatology, Okayama Univ.	In preparation
11/22/2013	Post cardiac arrest syndrome	Emergency and Critical care medicine, Keio Univ.	Calling for participants
2/22/2014	Eye disease	Ophthalmology, Nippon Medical school	Finished
7/1/2014	Acute myocardial infarction	Cardiology, National Center for Global Health and Medicine	Calling for participants
7/29/2014	Subarachnoid hemorrhage	Neurosurgery, Self Defense Medical College	Calling for participants [113]
8/1/2014	Lung transplantation	General thoracic surgery, Osaka Univ.	Calling for participants
10/27/2014	Retinal artery occlusion	Ophthalmology, Nippon Medical school	Calling for participants
7/3/2015	Type 2 diabetes mellitus	Tokyo Metropolitan Institute of Gerontology	Calling for participants

The department names are shown if they are available in the UMIN clinical trial database

## Conclusions

The number of original articles showing the effects of hydrogen are increasing yearly after 2007, and an extensive review of these articles are getting more and more difficult. Some of these articles, however, are a repetition of previous studies with insignificant novel findings. We suppose that almost all disease models and almost all modalities by which hydrogen is administered have been already examined. Large-scale controlled human studies and elucidation of molecular mechanisms underlying the effects of hydrogen are the next steps that must be pursued.

A dose–response effect of hydrogen is observed in drinking hydrogen-rich water [94, 97]. A similar dose–response effect is also observed in inhaled hydrogen gas [1, 17, 98]. However, when hydrogen concentrations in drinking water and in inhaled gas are compared, there is no dose–response effect. Hydrogen-rich water generally shows a more prominent effect than hydrogen gas, although the amount of hydrogen taken up by hydrogen water is ~100 times less than that given by hydrogen gas [11]. Gastric secretion of ghrelin may partly account for this difference [95]. Another factor that accounts for the effects of hydrogen is the temporal profile of hydrogen administration. Intermittent inhalation, but not continuous inhalation, of hydrogen is protective against a rat model of Parkinson’s disease, which is against a dose-responsiveness of hydrogen [11]. The prominent effects of molecular hydrogen in a variety of disease models, human diseases, treatment-associated pathologies, and pathophysiological conditions of plants have been disclosed in these 8 years, but unsolved conundrums still challenge us.
